# Epigenetic Regulation of Galectin-1 and Galectin-3 in Osteoporosis: A Pilot Study in Patients Undergoing Total Joint Arthroplasty

**DOI:** 10.3390/cells15121119

**Published:** 2026-06-21

**Authors:** Marina Russo, Gianluca Conza, Caterina Claudia Lepre, Gabriele Martin, Annalisa Itro, Adriano Braile, Gerardo Grossi, Nicoletta Tangredi, Michele D’Amico, Anca Hermenean, Maria Consiglia Trotta, Giuseppe Toro

**Affiliations:** 1 Department of Mental, Physical Health and Preventive Medicine, University of Campania “Luigi Vanvitelli”, 80138 Naples, Italy; marina.russo@unicampania.it; 2 School of Pharmacology and Clinical Toxicology, University of Campania “Luigi Vanvitelli”, 80138 Naples, Italy; 3 Multidisciplinary Department of Medical, Surgical and Dental Sciences, University of Campania “Luigi Vanvitelli”, 80138 Naples, Italy; gianluca.conza@studenti.unicampania.it (G.C.); gabriele.martin@studenti.unicampania.it (G.M.); annalisa.itro@studenti.unicampania.it (A.I.); adriano.braile@hotmail.it (A.B.); gergro88@gmail.com (G.G.); 4 Department of Experimental Medicine, University of Campania “Luigi Vanvitelli”, 80138 Naples, Italy; nicoletta0@hotmail.it (N.T.); michele.damico@unicampania.it (M.D.); mariaconsiglia.trotta2@unicampania.it (M.C.T.); 5 Department of Clinical Sciences and Translational Medicine, University of Rome “Tor Vergata”, 00133 Rome, Italy; 6 Faculty of Medicine, “Vasile Goldis” Western University of Arad, 310144 Arad, Romania; hermenean.anca@uvvg.ro

**Keywords:** osteoporosis, Galectin-1, Galectin-3, miR-22, miR-21

## Abstract

**Highlights:**

**What are the main findings?**

- Bones from osteoporotic patients showed reduced Gal-1 and increased miR-22 levels, together with increased Gal-3 and miR-21 levels.
- Serum analyses showed decreased Gal-1 and miR-22 and increased miR-21 in osteoporotic patients.

**What is the implication of the main finding?**

- These observed co-dysregulation patterns of miR-22, Gal-1, miR-21, and Gal-3 in osteoporotic bone may be associated with alterations in bone remodeling processes in osteoporosis.
- These Galectin–miRNA signatures could be putative molecular indicators potentially associated with osteoporosis-related bone alterations.

**Abstract:**

**Background:** Osteoporosis (OP) is a chronic disease characterized by decreased bone mass and altered microarchitecture, leading to bone fragility and fracture risk. To date, although carbohydrate-binding proteins Galectins 1 and 3 (Gal-1/Gal-3) have been implicated in bone metabolism, inflammation and aging, their levels and potential regulation by microRNAs (miRNAs) have not yet been investigated in OP. **Methods:** In this pilot study, 13 osteoporotic (OP) and 10 non-osteoporotic (NOP) patients, all undergoing hip or knee arthroplasty, were enrolled. Due to the unavailability of DXA measurements, OP classification was based on cortical bone ratio and distal femoral cortical index. Clinical parameters and blood samples were collected preoperatively, while bone biopsies were obtained intraoperatively. ELISA and qRT-PCR were used to quantify Gal-1, Gal-3, miR-22 and miR-21 in bones and sera. Correlations with clinical parameters were assessed. **Results:** Several OP biopsies exhibited a reduction in Gal-1 levels, whereas miR-22, Gal-3 and miR-21 were increased. Serum analysis revealed similar dysregulation patterns, with increased miR-21 and decreased Gal-1 and miR-22 levels in several OP patients. **Conclusions:** This pilot study suggests a putative association of Gal-1, Gal-3, and their previously reported related miRNAs with osteoporotic bone status, indicating their potential involvement in OP-related bone metabolism.

## 1. Introduction

Osteoporosis (OP) is defined by the World Health Organization (WHO) as a progressive systemic skeletal disease characterized by low bone mass and microarchitectural deterioration of bone tissue, with a consequent increase in bone fragility and susceptibility to fracture [1]. It represents a major public health concern, particularly in light of global population aging [2]. Osteoporotic fractures are a leading cause of disability and contribute substantially to healthcare costs worldwide [3], most commonly involving the hip, spine, shoulder, forearm, and pelvis [4,5,6]. Notably, the annual incidence of hip fracture is expected to increase to 6.3 million in 2050 [7]. OP is traditionally classified as primary or secondary [8]. Primary OP results from age- or menopause-related reductions in bone mass and mineralization, whereas secondary OP arises from specific underlying diseases or contributing factors [8,9]. The disease often remains clinically silent until a fragility fracture occurs [8], and the presence of an osteoporotic fracture markedly increases the risk of subsequent fractures [10]. Consequently, preventing second fracture represents the central goal of the current OP management strategies, which rely on specific healthcare pathways (i.e., fracture liaison services) and pharmacological therapy [11,12,13].

However, despite the effectiveness of multidisciplinary care models in reducing the risk of secondary fractures [12,13,14,15], diagnosing OP remains a clinical challenge, as the disease often progresses without clear symptoms, typically being diagnosed only after a fragility fracture has occurred. Although bone mineral density (BMD) derived from dual-energy X-ray absorptiometry (DXA) could miss some information on bone quality, microarchitectural deterioration and the multifactorial nature of fracture risk, it still represents the main diagnostic modality for OP [16,17]. Moreover, interpretation might also be complicated by artifacts such as osteoarthritis or vascular calcifications that could overestimate BMD. Bone turnover markers (BTMs), while potentially informative, add another layer of complexity due to their biological and analytical variability [18]. According to the Italian recommendations for OP diagnosis, a combination of the patient’s medical history, a thorough physical examination, standard thoracic and lumbar spine X-rays, BMD testing and relevant laboratory evaluations is necessary [8]. The risk assessment might be more carefully based on some risk charts like the FRAX (Fracture Risk Assessment Tool) or FRAHS (FRActure Health Search) [8].

Theoretically, an appropriate evaluation of OP diagnosis and treatment effectiveness might also be based on the evaluation of at least one bone resorption and one bone formation biomarker during the follow-up [19,20]. The most common bone formation biomarkers used in clinical practice are the N-terminal propeptide of type I procollagen (P1NP), osteocalcin (OC), and bone-specific alkaline phosphatase (ALP) [19,21]. The most widely used bone resorption markers include the C-terminal telopeptide of type I collagen (CTX), the N-terminal telopeptide of type I collagen (NTX), and tartrate-resistant acid phosphatase 5b (TRAP-5b). β-isomerized form of CTX (β-CTX-I) results from the degradation of type I collagen by osteoclasts and is one of the most sensitive markers for bone resorption [21]. Clinically, BTMs do not replace BMD measurements, but elevated BTMs are correlated with a faster BMD decline and an increased fracture risk [22]. BTM changes during therapy may precede BMD variations and reflect therapeutic response [23], potentially representing complementary tools for identifying high-risk individuals and monitoring therapeutic efficacy.

Given the growing interest in biomarkers that can provide early insights into bone metabolism, potentially enhancing the OP diagnosis and management, increasing interest has been directed toward other molecular regulators of skeletal homeostasis, including the carbohydrate-binding proteins Galectin-1 (Gal-1) and Galectin-3 (Gal-3) [24]. Particularly, a decline of bone Gal-1 levels mainly correlated with reduced mineral density, changes in trabecular organization and bone loss in aged mice [25]. Conversely, an increase in Gal-3 in osteoblasts has been associated with higher bone resorption and reduced bone mineralization [26,27,28]. However, the contribution of Gal-1 and Gal-3 to clinical OP remains insufficiently defined and, to our knowledge, their bone levels have not yet been evaluated in the OP population. Gals have been previously reported to be epigenetically regulated by microRNAs (miRNAs), small non-coding RNAs able to modulate protein expression [29,30,31]. A pivotal role for miRNAs has been described in promoting or inhibiting osteogenesis and modulating other mechanisms leading to bone loss [32]. Among these, miR-22 and miR-21 have been reported to be dysregulated in OP preclinical settings [33,34,35,36]. Moreover, miR-22 has been previously identified as an epigenetic regulator of Gal-1 [37,38,39,40], as well as miR-21, which seems to target Gal-3 [41,42,43,44] in clinical settings different from OP.

Nevertheless, no studies have yet investigated the co-dysregulation pattern of miR-22/Gal-1 and miR-21/Gal-3 in bone tissue from patients with OP. Therefore, their evaluations at the bone level may reveal novel mechanisms potentially contributing to OP and identify innovative therapeutic pathways. To this regard, the first aim of the present study was to quantify miR-22, Gal-1, miR-21, and Gal-3 in bone biopsies from OP patients undergoing hip or knee arthroplasty and to explore their potential associations with clinical characteristics. Moreover, since both circulating miR-22 and miR-21 have been previously reported to be dysregulated in the OP population [45,46,47,48,49,50] and a single clinical study has reported altered serum Gal-1 levels in male OP patients [51], we assessed circulating levels of these miRNAs and Gals. This additional analysis aimed to determine whether these molecules may be potentially associated with OP status and whether their circulating levels reflect tissue-level variations.

## 2. Materials and Methods

### 2.1. Study Design

This is an observational pilot study in which 23 patients undergoing total hip or knee arthroplasty (THA and TKA, respectively) were enrolled at the Unit of Orthopedics—University of Campania ‘Luigi Vanvitelli’ (Naples, Italy). The study was conducted based on the principles outlined in the Declaration of Helsinki and was approved by the Board of Reviewers of the University of Campania “Luigi Vanvitelli” (Prot. 0020791/I,31/07/2024). All enrolled patients provided informed consent to participate in the study. Inclusion and exclusion criteria were summarized in Table 1.

**Figure 1:**
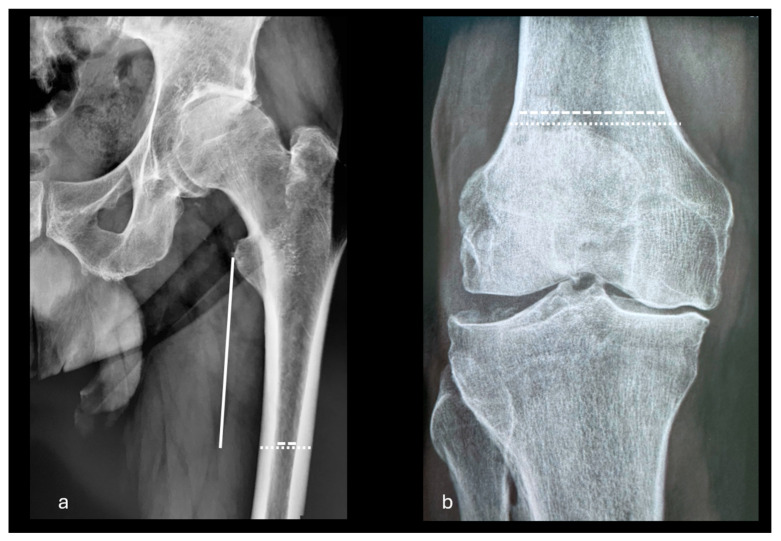
**Representative evaluation of the CBR and DFCI**. (**a**) The CBR is defined as the ratio of the endosteal diameter (dashed line) to the external diameter measured (dotted line) 10 cm below the lesser trochanter. (**b**) The DFCI is defined as the ratio of the endosteal diameter (dashed line) to the external diameter measured (dotted line) at the most distal point of the femoral diaphysis where the tangent line along the endosteal borders of the lateral diaphyseal cortices no longer remained continuous with the diaphysis. CBR: cortical bone ratio; DFCI: distal femoral cortical index.

Bone quality was retrospectively evaluated by assessing the cortical bone ratio (CBR) for the hips and the distal femoral cortical index (DFCI) for the knees, as previously reported (Figure 1) [53,54,55]. These panoramic radiographic indices have been investigated as surrogate markers of skeletal bone status in screening settings and have shown associations with reduced bone mineral density [53,54,55]. Accordingly, their use has been proposed as a supportive tool for assessment of OP risk, particularly in contexts where DXA is not available, as also reported in the literature and in screening-oriented recommendations [56,57].

A CBR over 0.48 and a DFCI over 1.10 were considered diagnostic for OP [53,54].

For each patient, clinical parameters and blood samples were collected before surgery, while bone biopsies were obtained during arthroplasty. In particular, for patients enrolled to undergo TKA, a median parapatellar approach was performed, and muscular biopsies were collected from detached fibers of the vastus medialis, whereas bone specimens were collected from the cut femoral condyles. For patients undergoing THA, muscular samples were collected from detached fibers of the gluteus medius, while bone biopsies were collected from the femoral heads removed during the procedure.

### 2.2. Collection of Blood Samples

Whole blood samples were collected prior to total hip or knee arthroplasty using sterile and vacutainer tubes. Within two hours of collection, samples were incubated for 30 min at 20 °C, then centrifuged at 3000 rpm for 15 min at 4 °C. The resulting sera were aliquoted and stored at -80° [58] for the determination of calcium (mg/dL), 25 hydroxyvitamin D (ng/mL), ALP (U/L) levels and circulating biomarkers.

### 2.3. Collection and Processing of Bone Biopsies

Once bone biopsies have been obtained during the THA or TKA, any residual soft tissue or blood contamination was removed from the biopsy with tweezers and scalpel, then performing several washes with ice-chilled sterile phosphate-buffered saline (PBS–Aurogene; Rome, Italy) before storing at −80 °C until use [59]. To perform tissue homogenization, biopsies were ground in liquid nitrogen with a pestle and mortar, obtaining 100 mg of bone reduced to a fine powder.

### 2.4. Enzyme-Linked Immunosorbent Assays (ELISAs)

For bone Gal-1 and Gal-3 determination, 50 mg of bone powder were processed with a pestle in RIPA buffer containing the Complete Protease Inhibitor Cocktail (Roche; Milan, Italy) and then incubated for 4 h at 4 °C with rocking [60]. Samples were then centrifuged at 14,000 rpm for 10 min 4 °C [61], by obtaining protein supernatants. Total protein concentration was measured by using the Bio-Rad Protein Assay (500-0006, Bio-Rad Laboratories; Milan, Italy) [62]. All samples were normalized to an equal total protein input (100 µg per assay), ensuring that ELISA measurements were performed under standardized and comparable conditions across all samples.

Bone and serum levels of Gal-1 (RK04523 Human Galectin 1/LGALS1 ELISA Kit, ABclonal—Woburn, MA, USA) and Gal-3 (EH0145 Human Galectin 3 ELISA Kit, FineTest; Palm Coast, FL, USA) were assessed by commercial ELISA kits, according to the manufacturer’s protocols.

### 2.5. Real-Time Polymerase Chain Reaction (qPCR) for miRNA Quantization

The isolation of miRNAs from bone biopsies was performed by using the miRNeasy Mini Kit (217004, Qiagen; Milan, Italy), starting from 50 mg of bone powder processed with a pestle in the suitable lysis buffer [59] and adding Syn-cel-miR-39 miScript miRNA Mimic 5 nM (2922723, Qiagen; Milan, Italy) as a spike-in control. This approach, widely accepted in circulating miRNA studies, was used to control for technical variability introduced during RNA isolation, reverse transcription, and qPCR amplification [63,64,65,66,67,68]. Serum miRNAs were isolated by using the miRNeasy Serum/Plasma Kit—microRNA Isolation (217184, Qiagen; Milan, Italy), adding miRNeasy Serum/Plasma Spike-In Control (219610, Qiagen; Milan, Italy). RNA concentration and quality were assessed by using a NanoDrop 2000c Spectrophotometer (Thermo Fisher Scientific; Waltham, MA, USA).

For the reverse transcription (RT) step, 20 ng of total RNA isolated from bone biopsies were converted to cDNA. The volume of RNA isolated from serum to reverse transcribed was calculated according to the protocol: First-Strand cDNA Synthesis (miRCURY LNA miRNA SYBR Green PCR—Exosomes, Serum/Plasma, and Other Biofluid Samples Handbook, Qiagen; Milan, Italy) as follows: Serum template RNA [μl] = Elution volume [μl]/Original sample volume [μl] * 16 [μl].

The RT phase was performed by using the miRCURY LNA RT Kit (339340, Qiagen: Milan, Italy), RNA Spike-In Kit, For RT (339390, Qiagen; Milan, Italy)—both according to the manufacturer’s protocols—and the Gene AMP PCR System 9700 (Applied Biosystems; Waltham, MA, USA). Particularly, an incubation of 60 min at 42 °C was followed by 5 min at 95 °C to heat inactivate the reverse transcriptase, before immediately cooling at 4 °C.

qPCR step was performed by using the miRCURY LNA SYBR Green PCR Kit (339346, Qiagen; Milan Italy), together with specific miRCURY LNA PCR Assay (339306, Qiagen; Milan, Italy) for hsa-miR22-3p (YP00204606, Qiagen; Milan, Italy), hsa-miR21-3p (YP00204302, Qiagen; Milan, Italy) and Cel-mir39-3p (YP00203952, Qiagen; Milan, Italy). This was used as an exogenous spike-in control for serum miRNA normalization due to its absence in human samples and ability to control for technical variability, whereas U6 snRNA was not used because of its nuclear origin, instability in extracellular fluids, and unreliability in serum-based analyses [69,70,71,72,73]. According to the “Quantitative, Real-Time PCR Using Individual miRCURY LNA RNA PCR Assays” (Qiagen; Milan, Italy), the triplicate qPCR reactions were carried out on the CFX96 Real-time System C1000 Touch Thermal Cycler (Biorad, Milan, Italy), by setting the following cycling conditions: 2 min at 95 °C for the PCR initial heat activation; a two-step cycling of 10 s at 95 °C for denaturation, 60 s at 56 °C for combined annealing/extension; followed by fluorescence data collection (repeated for 40 cycles). The 2^−ΔΔCt^ method was used for the relative quantization of gene expression.

### 2.6. Statistical Analysis

Levene’s test and the Shapiro–Wilk test were used to assess data homogeneity and distribution. A multivariate analysis of variance (MANOVA) was first performed to evaluate overall group differences between NOP and OP subjects across all biomarkers (bone/serum Gal-1, miR-22, Gal-3, miR-21). A multivariate analysis of covariance (MANCOVA) was subsequently conducted including age, sex, hyperlipidemia, and vitamin D deficiency as covariates. Univariate ANOVAs were then performed for each biomarker, and *p*-values were adjusted using the Benjamini–Hochberg False Discovery Rate (FDR) correction. For descriptive purposes, group comparisons between NOP and OP were also assessed using a t-test or Mann–Whitney U test, as appropriate. Effect sizes were calculated using Cohen’s d for parametric comparisons and Cliff’s delta for non-parametric comparisons. A *p*-value of *p* < 0.05 was considered statistically significant.

## 3. Results

At the Unit of Orthopedics—University of Campania ‘Luigi Vanvitelli’ (Naples, Italy), 23 patients (12 men and 11 women) with a mean age of 69.4 ± 7.5 years and undergoing THA (*N* = 13) or TKA (*N* = 10) were enrolled. All patients were diagnosed with stage IV osteoarthritis according to the Kellgren–Lawrence classification system [52]. Of these, 13 were diagnosed with OP (OP group), while 10 patients were non-osteoporotic and used as a control group (NOP group). Both groups showed similar age, sex distribution, body mass index (BMI) and calcium blood levels, while the OP group was characterized by a higher prevalence of vitamin D deficiency and hyperlipidemia (Table 2). The latter was significantly correlated with OP diagnosis (r = 0.47; *p* < 0.05) (Table S1).

### 3.1. Multivariate Analysis of Group Effects on Mediator Profiles

MANOVA revealed a significant overall effect of group (OP vs. NOP) on the combined mediator profile (Wilks’ Λ = 0.093, F (8,14) = 17.14, *p* < 0.01). The effect of the group remained significant after adjustment for age, sex, hyperlipidemia, and vitamin D deficiency in the MANCOVA model (Wilks’ Λ = 0.083, F (8,7) = 9.61, *p* < 0.01).

### 3.2. Bone miR-22/Gal-1 Co-Dysregulation

Firstly, a subgroup analysis was performed to assess potential differences in all analyzed mediators between hip- (THA) and knee-derived samples (TKA) within the NOP and OP groups. No statistically significant differences were observed within either anatomical subgroup, whereas the differences between NOP and OP remained consistently significant when comparing matched THA and TKA groups (Figure S1). Therefore, to ensure an adequate sample size for the analyses of this exploratory pilot study, all samples were pooled across anatomical sites.

**Figure 2:**
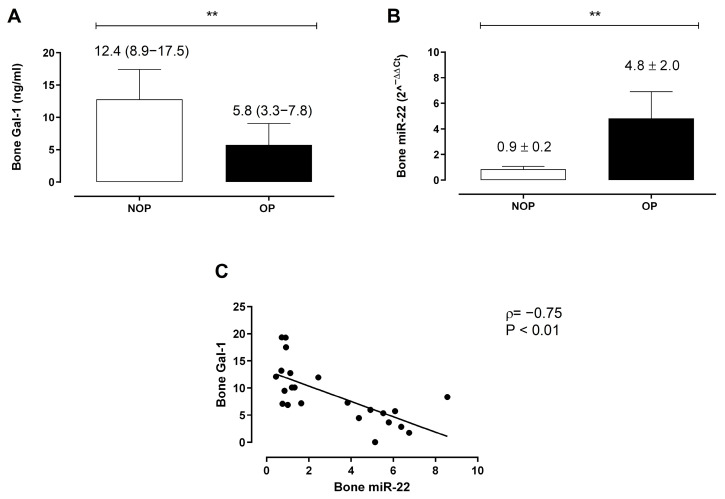
**Levels of Gal-1 and miR-22 in bone tissues.** (**A**) Bone Gal-1 levels (ng/mL) in non-osteoporotic (NOP, *N* = 10) and osteoporotic (OP, *N* = 13) patients. Data are reported as median and interquartile range (IQR); ** *p* < 0.01 (Mann–Whitney); FDR-corrected: q < 0.05; Cliff’s δ [NOP vs. OP] = −0.79. (**B**) Bone miR-22 levels (2^−ΔΔCt^) in NOP and OP patients. Data are reported as mean ± standard deviation (SD); ** *p* < 0.01 (T-test); FDR-corrected: q < 0.05; Cohen’s d = 2.67. (**C**) Spearman correlation between bone Gal-1 (ng/mL) with miR-22 levels (2^−ΔΔCt^); ρ = −0.75, *p* < 0.01. FDR-corrected: False Discovery Rate correction; Gal-1: Galectin-1; miR-22: microRNA-22; δ = Cliff’s delta; ρ = Spearman coefficient; r = Pearson coefficient.

Analyzing the Gal-1 levels in bone biopsies, we observed a significant down-regulation in osteoporotic patients (OP) compared to the control group (NOP) [NOP: 12.4 (8.9–17.5) ng/mL; OP: 5.8 (3.3–7.8) ng/mL, *p* < 0.01 vs. NOP] (Figure 2A).

Bone Gal-1 dysregulation in the OP group was paralleled by a significant increase in tissue miR-22 in OP compared to NOP patients (NOP: 0.9 ± 0.2 2^−ΔΔCt^; OP: 4.8 ± 2.0 2^−ΔΔCt^, *p* < 0.01 vs NOP) (Figure 2B).

The inverse trend shown by miR-22 and Gal-1 in bone biopsies was supported by their significant negative correlation (ρ = −0.75, *p* < 0.01) (Figure 2C).

Interestingly, miR-22 levels in bone tissues showed a significant mild positive correlation with hyperlipidemia (r = 0.50, *p* < 0.05) (Table 3).

### 3.3. Bone miR-21/Gal-3 Co-Dysregulation

**Figure 3:**
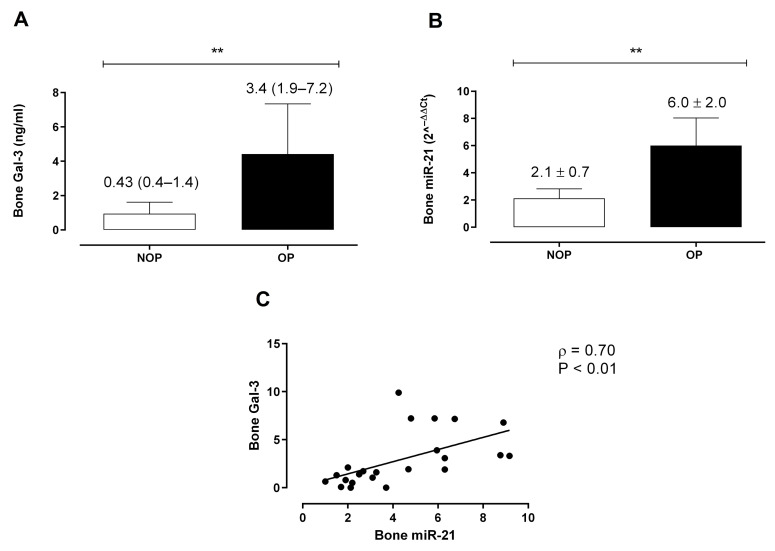
**Levels of Gal-3 and miR-21 in bone tissues.** (**A**) Bone Gal-3 levels (ng/mL) in non-osteoporotic (NOP, *N* = 10) and osteoporotic (OP, *N* = 13) patients. Data are reported as median and interquartile range (IQR); ** *p* < 0.01 (Mann–Whitney); FDR-corrected, q < 0.05; Cliff’s δ [NOP vs. OP] = 0.80. (**B**) Bone miR-21 levels (2^−ΔΔCt^) in NOP and OP patients. Data are reported as mean ± standard deviation (SD); ** *p* < 0.01 (T-test); FDR-corrected, q < 0.05; Cohen’s d = 2.56. (**C**) Spearman correlation between bone Gal-3 (ng/mL) with miR-21 levels (2^−ΔΔCt^); ρ = 0.70, *p* < 0.01. FDR-corrected: False Discovery Rate correction. Gal-3: Galectin-3. miR-21: microRNA-21. δ = Cliff’s delta. ρ = Spearman coefficient. r = Pearson coefficient.

The determination of Gal-3 in bone biopsies evidenced a significant up-regulation in OP compared to NOP patients [NOP: 0.43 (0.4–1.4) ng/mL; OP: 3.4 (1.9–7.2 ng/mL), *p* < 0.01 vs NOP] (Figure 3A).

Bone Gal-3 increase in OP patients was paralleled by a significant miR-21 increase in OP tissues compared to the NOP group (NOP: 2.1 ± 0.7 2^−ΔΔCt^; OP: 6.0 ± 2.0 2^−ΔΔCt^, *p* < 0.01 vs. NOP) (Figure 3B). Overall, miR-21 and Gal-3 in bone biopsies showed a significant strong positive correlation (ρ = 0.70, *p* < 0.01) (Figure 3C). The correlations between miR-21/Gal-3 and other clinical parameters are reported in Table 4.

### 3.4. Serum Levels of miR-22, Gal-1, miR-21 and Gal-3

**Figure 4:**
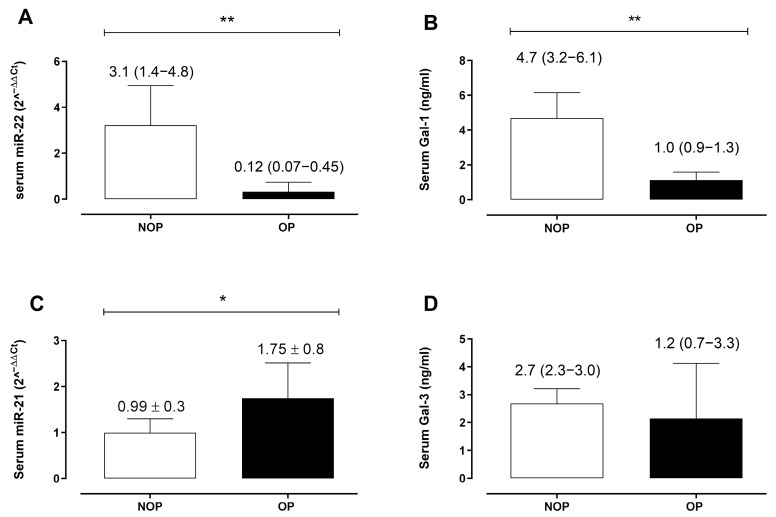
**Circulating levels of miR-22, Gal-1, miR-21 and Gal-3.** (**A**) Serum miR-22 levels (2^−ΔΔCt^) in non-osteoporotic (NOP, *N* = 10) and osteoporotic (OP, *N* = 13) patients. Data are reported as median and interquartile range (IQR); ** *p* < 0.01 (Mann–Whitney); FDR-corrected, q < 0.05; Cliff’s δ [NOP vs. OP] = −0.96. (**B**) Serum Gal-1 levels (ng/mL) in NOP and OP patients. Data are reported as median and IQR; ** *p* < 0.01 (Mann–Whitney); FDR-corrected, q < 0.05; Cliff’s δ [NOP vs. OP] = −1.00. (**C**) Serum miR-21 levels (2^−ΔΔCt^) in NOP and OP groups. Data are reported as mean ± standard deviation (SD); * *p* < 0.05 (T-test); FDR-corrected, q < 0.05; Cohen’s d = 1.70. (**D**) Serum Gal-3 levels (ng/mL) in NOP and OP patients. Data are reported as median and IQR; *p* > 0.05 (Mann–Whitney); FDR-corrected, q > 0.05; Cliff’s δ [NOP vs. OP] = −0.43. FDR-corrected: False Discovery Rate correction. Gal-1: Galectin-1. Gal-3: Galectin-3. miR-21: microRNA-21. miR-22: microRNA-22. δ = Cliff’s delta. ρ = Spearman coefficient. r = Pearson coefficient.

The analysis of circulating miRNAs and Gals evidenced a significant dysregulation in OP patients of serum miR-22, Gal-1 and miR-21 (Figure 4).

Particularly, serum miR-22 was significantly downregulated in OP patients [NOP: 3.1 (1.4–4.8) 2*^−^*^ΔΔCt^; OP: 0.12 (0.07–0.45) 2*^−^*^ΔΔCt^*, p* < 0.01 vs. NOP] (Figure 4A). The same trend was evidenced by the analysis of serum Gal-1 levels, significantly downregulated in the OP group [NOP: 4.7 (3.2–6.1) ng/mL; OP: 1.0 (0.9–1.3) ng/mL*, p* < 0.01 vs. NOP] (Figure 4B). The correlations between serum miR-22/Gal-1 with clinical parameters and circulating mediators are reported in Table S2. Regarding the miR-21/Gal-3 dysregulation, serum miR-21 was significantly increased in OP patients (NOP: 0.99 ± 0.3 2*^−^*^ΔΔCt^; OP: 1.75 ± 0.8 2*^−^*^ΔΔCt^, *p* < 0.05 vs. NOP) (Figure 4C). Conversely, serum Gal-3 levels were not significantly dysregulated in OP patients compared to NOP [NOP: 2.7 (2.3–3.0) ng/mL; OP: 1.2 (0.7–3.3) ng/mL*, p* > 0.05 vs. NOP] (Figure 4D). Furthermore, serum miR-21 levels showed a significant positive correlation with BMI (r = 0.69, *p* < 0.01) (Table S3).

### 3.5. Univariate Analyses of Biomarker Differences Between Groups

Univariate ANOVA demonstrated significant differences between groups for bone Gal-1 (F = 18.01, *p* = 0.00036), bone miR-22 (F = 35.33, *p* < 0.01), bone Gal-3 (F = 13.43, *p* = 0.01), bone miR-21 (F = 33.25, *p* < 0.01), serum Gal-1 (F = 69.54, *p* < 0.01), serum miR-22 (F = 34.74, *p* < 0.01), and serum miR-21 (F = 6.87, *p* < 0.05), whereas serum Gal-3 was not significantly different between groups (F = 0.69, *p* > 0.05).

After FDR correction, all biomarkers remained significant (q < 0.05), except serum Gal-3 (q > 0.05).

## 4. Discussion

To date, the pharmacological options available for OP treatment present some limitations, such as the partial skeletal microarchitecture restoration, rare but serious side effects of long-term antiresorptive drugs, and the high cost and the limited duration of anabolic therapies [74,75,76]. Therefore, the identification of novel OP molecular targets and their epigenetic regulation underlying bone remodeling could improve current knowledge and contribute to the development of more effective therapeutic strategies.

The best-characterized pathways implicated in OP include RANK/RANKL/OPG, regulating osteoclast differentiation and survival, and the estrogen–estrogen receptor (ER) signaling pathway, mediating bone health and repair [77,78,79]. Both are targets of pharmacological treatments, respectively Denosumab and Selective Estrogen Receptor Modulators (SERMs) [78,80] and are modulated by epigenetically miRNAs (miR-217, miR-503, miR-17/20a, miR-155 and miR-183 for RANK/RANKL/OPG; miR-133, miR-29a-3p, miR-93-5p, miR-486 and miR-30b for ER)[81,82,83,84,85,86,87]. 

Besides Wnt/β-catenin and SOST signaling [88,89,90,91], Gal-1 and Gal-3 have recently emerged as regulators of osteoblast/osteoclast functions [24]. These Gals are broadly implicated in processes relevant to OP, including inflammation, aging, immunity and tissue repair [24,92,93]. Gal-3 shows the highest bone specificity, expressed by osteoblasts and osteoclasts [94,95,96,97], while both Gal-1 and Gal-3 have been reported to interact with human factor VIII (FVIII) [98], involved in bone mineralization and remodeling [99,100]. Gal-1 and Gal-3 are constitutively expressed and secreted by bone marrow mesenchymal stem cells (BMSCs) [101,102,103], which can differentiate into osteoblasts and modulate bone formation [104]. However, to our knowledge, Gal-1 and Gal-3 bone levels have been assessed in only a few preclinical studies [24].

Our pilot study evidenced, for the first time, a significant decrease in Gal-1 in bone biopsies from osteoporotic patients, consistent with trabecular microarchitecture alterations and reduced BMD reported in aged Gal-1 knockout (KO) mice [25]. Gal-1 supplementation enhanced osteoblast differentiation in BMSCs derived from Gal-1 KO mice [25], while recombinant Gal-1 reduced bone-resorptive activity in both murine and human osteoclasts differentiated from peripheral blood mononuclear cells (PBMCs) [105]. Conversely, a single study reported a detrimental effect of Gal-1 treatment in human BMSCs, via reduced OC [106].

Regarding epigenetic modulation, several studies reported miR-22 as a specific Gal-1 regulator in several clinical settings [38,39,40,41]. Our pilot study evidenced a higher expression of miR-22 in osteoporotic bones, consistent with previously reported inhibitory effects on osteoblast differentiation in vitro and in aged mice [34,35]. Notably, we observed an inverse correlation between miR-22 and Gal-1 levels in osteoporotic bone. Bone miR-22 expression showed a significant association with hyperlipidemia, a known risk factor for osteoporosis [107,108,109]. Given that miR-22 is involved in lipid and metabolic homeostasis and has been reported to promote adipogenesis [110,111], the observed association between bone miR-22 expression and hyperlipidemia in our osteoporotic cohort may reflect a link between miR-22-associated metabolic dysregulation and alterations in bone remodeling observed in dyslipidemic conditions [112].

The analysis of Gal-3 and miR-21 in osteoporotic tissues revealed a significant up-regulation of both mediators, which showed a positive correlation, in agreement with previous observations in non-skeletal contexts [42,43,44,45]. Particularly, our results are consistent with a single clinical study evidencing a higher expression of bone miR-21 in osteoporotic patients [113]. This evidence is supported by several findings reporting an association between miR-21 expression and osteoclastogenic activity in vitro and in vivo [114,115,116]. Conversely, other preclinical evidence has suggested a beneficial role for miR-21 in stimulating osteoblast differentiation and activity, ultimately enhancing matrix mineralization [117,118,119]. Also, the Gal-3 increase detected in osteoporotic bone biopsies is in line with different preclinical OP studies. Indeed, Gal-3 exposure has been reported to be associated with changes in osteoclast differentiation in experimental models [28]. Gal-3 treatment in human fetal osteoblasts has been associated with alterations in osteoblast and osteoclast activity in experimental settings [29]. A protective effect of Gal-3 silencing was also observed in aged Gal-3 KO mice, which showed increased osteoblastogenesis and preserved—or even enhanced—bone mass [120]. Conversely, in the same in vivo Gal-3 KO model, Gal-3 deficiency led to a higher osteoclast number, paralleled by a reduced trabecular bone volume [121].

Although the bone sampling heterogeneity may have influenced our results (since the analyzed specimens differ in embryonic origin, trabecular and cortical bone composition, mechanical loading environment, and cellular and metabolic activity, potentially introducing variability in cortical thickness, porosity, and bone quality parameters [122,123,124]), a statistically powered site-specific analysis was not feasible due to the limited sample size of this pilot cohort. Nevertheless, preliminary subgroup analysis did not indicate relevant differences in the expression trends of the analyzed markers between hip- and knee-derived samples. Overall, our findings suggest that the observed co-dysregulation patterns involving miR-22, miR-21, Gal-1, and Gal-3 in osteoporotic bone and serum may reflect coordinated alterations in bone remodeling-related molecular profiles in OP. This supports the exploratory evaluation of circulating miRNAs as non-invasive indicators of systemic molecular changes related to bone metabolism. This is due to their high detectability, stability, and distinct spatiotemporal expression patterns in body fluids [125]. Notably, specific serum miRNAs have been associated with the long-term efficacy of Denosumab in postmenopausal osteoporotic patients [126] and with the risk of atypical fractures in patients receiving long-term BPs [127], or have been recently proposed as exploratory molecular indicators in OP contexts [48,128].

In this context, serum miR-22 levels have been found downregulated in our OP cohort, in line with previous studies [46,129,130], showing a discordant pattern from its bon expression. To this regard, several studies showed that the release of miRNAs into the body fluids is influenced by selective secretion mechanisms, cellular turnover, and the packaging into extracellular vesicles or protein complexes [71,131]. Therefore, the divergence between tissue and serum miR-22 levels may reflect predominant tissue-associated regulation in bone, potentially influenced by systemic release dynamics.

Regarding serum miR-21, its levels showed a similar trend to bone expression in osteoporotic patients, in line with previous clinical studies [35,47,49,50,113]. Although our results do not show the elevation of serum miR-21 levels in osteoporotic females previously described [48,132], these were found to correlate with clinical variables such as BMI.

In the context of non-invasive biomarkers, also Gal-1 and Gal-3 can be detected in body fluids, exhibiting both cellular and extracellular expression [133,134] and have been investigated as potential biomarkers for several pathological conditions [135,136,137]. Since circulating Gal-1 and Gal-3 pattern remain poorly characterized in OP, we evaluated a possible dysregulation of both serum Gal-1 and Gal-3 in our OP cohort. In line with a single study reporting decreased Gal-1 serum levels in osteoporotic males [52], we evidence for the first time a significant reduction in serum Gal-1 in osteoporotic population with a balanced sex distribution, showing a similar direction of change compared with bone levels. Conversely, serum Gal-3 was not found significantly altered in our clinical OP setting, not showing a pattern fully consistent with bone expression.

In conclusion, our evidence highlights a co-dysregulation pattern of Gals-miRs in OP, reporting bone Gal-1 and Gal-3 as altered in osteoporotic patients together with miR-22 and miR-21. However, no causal or direct regulatory relationships can be inferred from the present observational data, and thus these interactions should be interpreted as putative associations rather than mechanistic regulatory axes. Altogether, our findings provide initial integrated observational evidence that Gals-miRs co-dysregulation patterns may be associated with bone homeostasis-related molecular changes.

To date, the most common approach used at the preclinical levels to reduce miRNA activity is represented by antagomiRs directed against miR-148a, miR-31a-5p, and miR-103a, demonstrating suppression of bone resorption and enhancement of bone mass [93,94,95,96]. Regarding Gal-1 and Gal-3 inhibition, several compounds (GB1211, GR-MD-02, GB0139, Modified Citrus Pectin and OTX008) are currently under clinical investigation in non-skeletal diseases, suggesting their translational potential. These ongoing trials inform future exploratory investigations in bone-related conditions such as OP, once safety profiles are established. Therefore, these antagomiRs and Gal-inhibitors could represent experimental tools for modulating bone-associated molecular alterations, rather than established therapeutic strategies for OP.

Moreover, mir-22, miR-21 and Gal-1 serum patterns correlated with osteoporotic condition, suggesting a potential role for miR-21 and Gal-1 as exploratory molecular indicators potentially associated with OP, as their levels consistently reflect bone tissue dysregulation.

Nevertheless, the findings from our pilot study have some limitations. Importantly, the limited sample size could introduce potential bias and restrict the robustness and the generalizability of the results. Indeed, although altered levels of Gal-1, Gal-3, miR-21, and miR-22 were observed in several osteoporotic patients, these changes were not uniformly detected across the entire cohort, highlighting the exploratory nature of the study and the need for validation in larger patient populations. Additionally, we acknowledge that the absence of DXA-based measurements represents an important limitation of the present study. Although DXA is the gold standard for OP diagnosis, it was not available in the study population; therefore, subjects were classified as OP or NOP using validated radiographic indices (CBR and DFCI) applied according to established thresholds. While these indices are widely used for screening purposes and have demonstrated clinical utility in identifying reduced bone quality, they do not provide a direct equivalent to DXA-confirmed OP. For this reason, our classification should be interpreted as a radiographic surrogate definition of bone status within an exploratory study framework rather than a definitive clinical diagnosis. Future studies incorporating both radiographic assessment and DXA measurements will be essential to further validate and strengthen the present findings.

Serum biomarker alterations may still be influenced, at least in part, by systemic metabolic and inflammatory conditions such as hyperlipidemia and vitamin D deficiency. Although these variables were included as covariates in the multivariate analysis, their known biological associations with inflammatory pathways, macrophage activation, and bone metabolism [138,139,140,141] may contribute to inter-individual variability in circulating markers.

Consequently, further studies with a larger cohort of osteoporotic patients are required to validate the co-dysregulation of miR-22/Gal-1 and miR-21/Gal-3 hypothesized here as potential novel molecular indicators associated with OP and to clarify the correlation between OP and the new systemic biomarkers evaluated in this study.

## Figures and Tables

**Table 1:** **Description of inclusion and exclusion criteria used in the study.**

Inclusion Criteria	Exclusion Criteria
Diagnosis of grade end-stage osteoarthritis (III to IV according to Kellgren-Lawrence [52]) with surgical indication of performing a total joint arthroplasty	Concomitant diseases that negatively affect the quality of the musculoskeletal system (e.g., rheumatoid arthritis, connective tissue diseases, dystrophies, decompensated endocrinopathies)
Age > 60 years	Diagnosis of carcinoma
Ability to understand the study protocol and to sign the written informed consent	

**Table 2:** **Patient characteristics.**

Patients Enrolled, n	23
Males; *N* (%)	12 (52%)
Females; *N* (%)	11 (48%)
THA vs. TKA; *N* (%)	13 (57%) vs. 10 (43%)
	
NOP; *N* (%)	10 (43%)
OP; *N* (%)	13 (57%)
Males vs. Females—NOP; *N* (%)	5 (50%) vs. 5 (50%)
Males vs. Females—OP; *N* (%)	6 (46%) vs. 7 (54%)
	
Age, Years; (Mean ± SD)	69.4 ± 7.5
Age—NOP, Years; (Mean ± SD)	67.3 ± 8.4
Age—OP, Years; (Mean ± SD)	71.2 ± 6.9
	
BMI, Kg/m^2^ (Mean ± SD)	31.7 ± 5.4
BMI—NOP, Kg/m^2^ (Mean ± SD)	31.6 ± 6.0
BMI—OP, Kg/m^2^ (Mean ± SD)	31.8 ± 5.1
	
Hypovitaminosis D; *N* (%)	16 (69%)
Hypovitaminosis D—OP; *N* (%)	9 (56%)
Calcium Blood Levels; (Mean ± SD)	9.2 ± 0.5 mg/dL
Smoking; *N* (%)	8 (35%)
ALP; U/L (Mean ± SD)	90 ± 33
Hypertension; *N* (%)	17 (74%)
Diabetes; *N* (%)	10 (43%)
Hyperlipidemia; *N* (%)	11 (48%)
Hyperlipidemia—OP; *N* (%)	8 (72%)
Lung Diseases; *N* (%)	6 (26%)
Autoimmune Diseases; *N* (%)	6 (26%)

Hypovitaminosis D: 25 hydroxyvitamin D ≤ 20 ng/mL. Normal range for calcium blood levels: 8.8—10.4 mg/dL. Normal blood range for ALP: 44–147 U/L. BMI: body mass index. NOP: non-osteoporotic. OP: osteoporotic. ALP: alkaline phosphatase. *N*: number. SD: standard deviation. %: percentage.

**Table 3:** **Pearson correlations between bone Gal-1/miR-22 with clinical parameters.**

	Bone Gal-1	Bone miR-22
Gender	ρ = −0.40; *p* = 0.06	r = 0.03; *p* = 0.89
Age	ρ = 0.11; *p* = 0.61	r = 0.26; *p* = 0.25
Blood calcium levels	ρ = −0.42; *p* = 0.08	r = 0.16; *p* = 0.46
Hypovitaminosis D	ρ = −0.14; *p* = 0.54	r = 0.28; *p* = 0.21
BMI	ρ = −0.50; *p* = 0.07	r = 0.43; *p* = 0.07
Smoking	ρ = −0.47; *p* = 0.08	r = 0.41; *p* = 0.06
ALP	ρ = 0.27; *p* = 0.25	r = 0.06; *p* = 0.80
Hypertension	ρ = −0.24; *p* = 0.28	r = 0.06; *p* = 0.79
Diabetes	ρ = 0.22; *p* = 0.32	r = −0.34; *p* = 0.17
Hyperlipidemia	ρ = −0. 22; *p* = 0.32	r = 0.50; *p* < 0.05
Lung Diseases	ρ = −0.27; *p* = 0.23	r = 0.15; *p* = 0.51
Autoimmune Diseases	ρ = −0.27; *p* = 0.22	r = 0.11; *p* = 0.62

BMI: body mass index. ALP: alkaline phosphatase. ρ: Spearman coefficient. r: Pearson coefficient.

**Table 4:** **Correlations between bone Gal-3/miR-21 with clinical parameters.**

	Bone Gal-3	Bone miR-21
Gender	ρ = 0.01; *p* = 0.95	r = −0.10; *p* = 0.99
Age	ρ = 0.19; *p* = 0.40	r = 0.18; *p* = 0.43
Blood Calcium Levels	ρ = 0.05; *p* = 0.80	r = 0.26; *p* = 0.23
Hypovitaminosis D	ρ = −0.01; *p* = 0.97	r = −0.13; *p* = 0.56
BMI	ρ = 0.14; *p* = 0.54	r = 0.39; *p* = 0.07
Smoking	ρ = 0.18; *p* = 0.42	r = 0.21; *p* = 0.33
ALP	ρ = −0.19; *p* = 0.43	r = 0.08; *p* = 0.72
Hypertension	ρ = −0.15; *p* = 0.49	r = −0.07; *p* = 0.74
Diabetes	ρ = −0.21; *p* = 0.35	r = −0.09; *p* = 0.68
Hyperlipidemia	ρ = 0.14; *p* = 0.52	r = 0.42; *p* = 0.06
Lung Diseases	ρ = 0.21; *p* = 0.34	r = 0.16; *p* = 0.54
Autoimmune Diseases	ρ = −0.11; *p* = 0.62	r = 0.15; *p* = 0.49

BMI: body mass index. ALP: alkaline phosphatase. ρ = Spearman coefficient. r: Pearson coefficient.

## Data Availability

All data relevant to the study are included within the article and its Supplementary Materials.

## Supplementary Materials

The following supporting information can be downloaded at: https://www.mdpi.com/article/10.3390/cells15121119/s1, Table S1: Pearson correlations between OP with clinical parameters; Table S2: Correlations between serum miR-22/Gal-1 with clinical parameters and circulating mediators; Table S3: Correlations between serum miR-21/Gal-3 with clinical parameters and circulating mediators. Figure S1: Bone levels of Gal-1, miR-22, Gal-3 and miR-21 according to anatomical site (hip versus knee) and OP status.

## Abbreviations

The following abbreviations are used in this manuscript:

β-CTX-I	β-isomerized form of CTX
ρ	Spearman Coefficient
ALP	Alkaline phosphatase
AntagomiRs	anti-miRNA oligonucleotides
BPs	Bisphosphonates
BMD	Bone Mineral Density
BMI	Body Mass Index
BMSCs	Bone Marrow Mesenchymal Stem Cells
BTMs	Bone Turnover Markers
CBR	Cortical Bone Ratio
CTX	C-terminal telopeptide of type I collagen
DFCI	Distal femoral cortical index
DXA	Dual-energy X-ray absorptiometry
ELISA	Enzyme-linked immunosorbent assay
ER	Estrogen Receptor
FRAHS	FRActure Health Search
FRAX	Fracture Risk Assessment Tool
FVIII	Human Factor VIII
Gal-1	Galectin 1
Gal-3	Galectin 3
IQR	Interquartile Range
KO	Knock-out
miRNAs	microRNAs
NF-κB	nuclear kappa-light-chain-enhancer of activated B cells
NTX	the N-terminal telopeptide of type I collagen
OC	Osteocalcin
OP	Osteoporosis
OPG	Osteoprotegerin
P1NP	N-terminal propeptide of type I procollagen
PBMCs	Peripheral blood mononuclear cells
PBS	Phosphate-buffered saline
PTH	Parathyroid hormone
qPCR	Real-time polymerase chain reaction
r	Pearson Coefficient
RANK	Receptor activator of NF-κB
RANKL	Receptor activator of NF-κB ligand
RT	Reverse Transcription
SD	Standard Deviation
SERMS	Selective Estrogen Receptor Modulators
SOST	Sclerostin
THA	Total Hip Arthroplasty
TKA	Total Knee Arthroplasty
TRAP-5b	Tartrate-resistant acid phosphatase 5b
WHO	World Health Organization
