# Supplementary material for: Epigenetic Regulation of Galectin-1 and Galectin-3 in Osteoporosis: A Pilot Study in Patients Undergoing Total Joint Arthroplasty
Source: Cells. 2026 Jun 21;15(12):1119. doi: 10.3390/cells15121119 (PMC13296777; doi:10.3390/cells15121119)
Supplement: Supplementary file 1 [file cells-15-01119-s001.zip › cells-4341108-supplementary.pdf]

cells

Article

Epigenetic Regulation of Galectin-1 and Galectin-3 in Osteoporosis: A Pilot Study in Patients Undergoing Total Joint Arthroplasty

Marina Russo [1,2,†], Gianluca Conza [3,†], Caterina Claudia Lepre [4,*], Gabriele Martin [3], Annalisa Itro [3], Adriano Braile [3,5], Gerardo Grossi [3], Nicoletta Tangredi [4], Michele D'Amico [4], Anca Hermenean [6], Maria Consiglia Trotta [4,‡] and Giuseppe Toro [3,*,‡]

[1] Department of Mental, Physical Health and Preventive Medicine, University of Campania "Luigi Vanvitelli", 80138 Naples, Italy; marina.russo@unicampania.it
[2] School of Pharmacology and Clinical Toxicology, University of Campania "Luigi Vanvitelli", 80138 Naples, Italy
[3] Multidisciplinary Department of Medical, Surgical and Dental Sciences, University of Campania "Luigi Vanvitelli", 80138 Naples, Italy; gianluca.conza@studenti.unicampania.it (G.C.); gabriele.martin@studenti.unicampania.it (G.M.); annalisa.itro@studenti.unicampania.it (A.I.); adriano.braile@hotmail.it (A.B.); gergro88@gmail.com (G.G.)
[4] Department of Experimental Medicine, University of Campania "Luigi Vanvitelli", 80138 Naples, Italy; nicoletta0@hotmail.it (N.T.); michele.damico@unicampania.it (M.D.); mariaconsiglia.trotta2@unicampania.it (M.C.T.)
[5] Department of Clinical Sciences and Translational Medicine, University of Rome "Tor Vergata", 00133 Rome, Italy
[6] Faculty of Medicine, "Vasile Goldis" Western University of Arad, 310144 Arad, Romania; hermenean.anca@uvvg.ro
* Correspondence: caterinaclaudia.lepre@unicampania.it (C.C.L.); giuseppe.toro@unicampania.it (G.T.)
† These authors contributed equally to this work.
‡ These authors also contributed equally to this work.

SUPPLEMENTARY MATERIALS

Academic Editor: Kin Hing William Lau

Received: 11 May 2026
Revised: 17 June 2026
Accepted: 19 June 2026
Published: 21 June 2026

Copyright: © 2026 by the authors. Licensee MDPI, Basel, Switzerland. This article is an open access article distributed under the terms and conditions of the Creative Commons Attribution (CC BY) license.

Supplementary Tables

Table S1. Pearson correlations between OP with clinical parameters

	OP diagnosis
Gender	r = 0.14; P = 0.53
Age	r = 0.28; P = 0.23
Blood calcium levels	r = 0.36; P = 0.09
Hypovitaminosis D	r = 0.09; P = 0.72
BMI	r = 0.09; P = 0.69
Smoking	r = -0.19; P = 0.48
ALP	r = -0.09; P= 0.71
Hypertension	r = -0.06; P = 0.79
Diabetes	r = -0.16; P = 0.48
Hyperlipidemia	r = 0.47; P = 0.02
Lung Diseases	r = 0.06; P = 0.79
Autoimmune Diseases	r = 0.06; P = 0.79

BMI: body mass index; OP: osteoporosis; ALP: alkaline phosphatase; r: Paerson coefficient.

Table S2. Correlations between serum miR-22/Gal-1 with clinical parameters and circulating mediators

	Serum miR-22	Serum Gal-1
Gender	ρ = -0.17; P = 0.46	ρ = -0.15; P = 0.94
Age	ρ = -0.29; P = 0.18	ρ = -0.41; P = 0.06
Blood calcium levels	ρ = -0.37; P = 0.09	ρ = 0.02; P = 0.93
Hypovitaminosis D	ρ = 0.03; P = 0.89	ρ = -0.16; P = 0.48
BMI	ρ = -0.40; P = 0.08	ρ = -0.47; P = 0.09
Smoking	ρ = -0.31; P = 0.16	ρ = -0.24; P = 0.28
ALP	ρ = -0.18; P = 0.45	ρ = -0.12; P = 0.61
Hypertension	ρ = 0.11; P = 0.61	ρ = 0.15; P = 0.50
Diabetes	ρ = 0.21; P = 0.36	ρ = 0.17; P = 0.44
Hyperlipidemia	ρ = -0.40; P = 0.08	ρ = -0.48; P = 0.10
Lung Diseases	ρ = -0.15; P = 0.51	ρ = -0.25; P = 0.25
Autoimmune Diseases	ρ = -0.04; P = 0.85	ρ = -0.11; P = 0.62
Bone Gal-1	ρ = 0.61; P < 0.01	ρ = 0.54; P < 0.01
Bone miR-22	ρ = -0.73; P < 0.001	ρ = -0.72; P < 0.001
Serum Gal-1	ρ = 0.76; P < 0.001	-

BMI: body mass index; OP: osteoporosis; ALP: alkaline phosphatase; Gal-1: Galectin 1; miR-22: microRNA 22; ρ = Spearman coefficient.

https://doi.org/10.3390/cells15121119

Table S3. Correlations between serum miR-21/Gal-3 with clinical parameters and circulating mediators

	Serum miR-21	Serum Gal-3
Gender	r = 0.37; P = 0.08	ϱ = -0.08; P = 0.71
Age	r = 0.26; P = 0.25	ϱ = -0.29; P = 0.18
Blood calcium levels	r = 0.24; P = 0.27	ϱ = -0.24; P = 0.28
Hypovitaminosis D	r = 0.06; P = 0.78	ϱ = 0.25; P = 0.27
BMI	r = 0.69; P < 0.01	ϱ = -0.23; P = 0.21
Smoking	r = -0.19; P = 0.40	ϱ = 0.14; P = 0.54
ALP	r = -0.07; P = 0.76	ϱ = -0.17; P = 0.48
Hypertension	r = -0.19; P = 0.41	ϱ = 0.21; P = 0.34
Diabetes	r = -0.35; P = 0.11	ϱ = 0.13; P = 0.57
Hyperlipidemia	r = 0.36; P = 0.10	ϱ = 0.10; P = 0.65
Lung Diseases	r = -0.02; P = 0.92	ϱ = -0.02; P = 0.95
Autoimmune Diseases	r = 0.27; P = 0.23	ϱ = 0.32; P = 0.15
Bone Gal-3	r = 0.33, P = 0.12	ϱ = -0.60; P < 0.01
Bone miR-21	r = 0.51, P = < 0.05	ϱ = -0.19, P = 0.38
Serum Gal-3	r = -0.03, P = 0.86	-

BMI: body mass index; OP: osteoporosis; ALP: alkaline phosphatase; Gal-3: Galectin 3; miR-21: microRNA 21; ϱ = Spearman coefficient; r: Paerson coefficient.

https://doi.org/10.3390/cells15121119

Supplementary Figures

Figure S1. Bone levels of Gal-1, miR-22, Gal-3 and miR-21 according to anatomical site (hip versus knee) and OP status

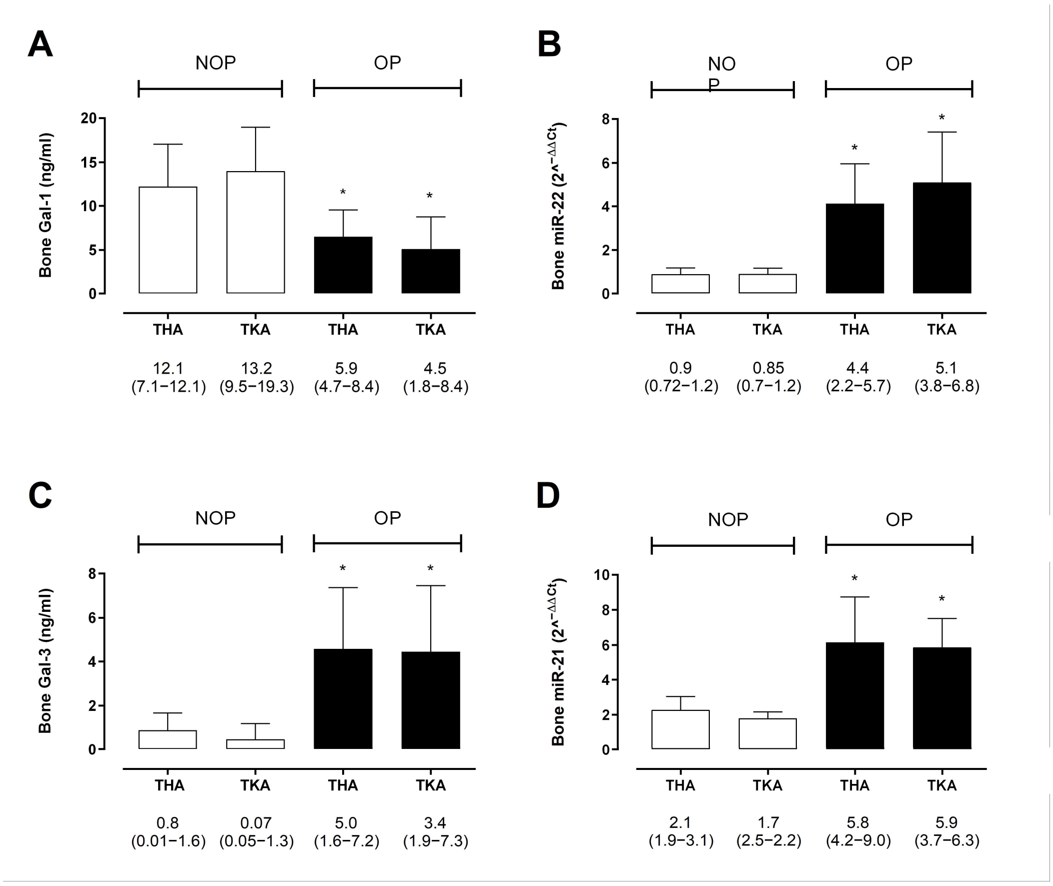

Bone levels of (A) Gal-1 (ng/ml), (B) miR-22 ($2^{\wedge-\Delta\Delta Ct}$), (C) Gal-3 (ng/ml) and (D) miR-21 ($2^{\wedge-\Delta\Delta Ct}$) in NOP (non-osteoporotic, N = 10) and OP (osteoporotic, N = 13) subjects subdivided in THA (patients undergoing total hip arthroplasty, NOP: N = 7; OP: N = 6) and TKA (patients undergoing total knee arthroplasty, NOP: N = 3; OP: N = 7) groups. Data are reported as median and interquartile range (IQR); * $P < 0.05$ vs NOP, same total arthroplasty (Mann-Whitney); FDR-corrected: $q < 0.05$. FDR-corrected: False Discovery Rate correction; Gal-1: Galectin-1; Gal-3: Galectin-3; miR-21: microRNA-21; miR-22: microRNA-22;

https://doi.org/10.3390/cells15121119
